# Singular Cause of Death!

**Published:** 1848-07

**Authors:** 


*Singular Cause of Death!*—The municipal regulations of the city of New York, require that the physician attending every case of disease, which proves fatal, shall deliver, in writing, a statement of the cause of death, for registration. The following has been handed us, by a friend, who states that it is a literal transcript of the cause of the death of a patient, as registered, in the language of the medical attendant:—

" *This woman was died, because she did die, and she was die of vickness, and she could not live.* *DR. VANDERHIDEN.*

